# Tiger sharks can connect equatorial habitats and fisheries across the Atlantic Ocean basin

**DOI:** 10.1371/journal.pone.0184763

**Published:** 2017-09-19

**Authors:** André S. Afonso, Ricardo Garla, Fábio H. V. Hazin

**Affiliations:** 1 Departamento de Pesca e Aquicultura, Universidade Federal Rural de Pernambuco, Recife, Pernambuco, Brazil; 2 Departamento de Botânifca Ecologia e Zoologia, Universidade Federal do Rio Grande do Norte, Natal, Rio Grande do Norte, Brazil; University of California Davis, UNITED STATES

## Abstract

Increasing our knowledge about the spatial ecology of apex predators and their interactions with diverse habitats and fisheries is necessary for understanding the trophic mechanisms that underlie several aspects of marine ecosystem dynamics and for guiding informed management policies. A preliminary assessment of tiger shark (*Galeocerdo cuvier*) population structure off the oceanic insular system of Fernando de Noronha (FEN) and the large-scale movements performed by this species in the equatorial Atlantic Ocean was conducted using longline and handline fishing gear and satellite telemetry. A total of 25 sharks measuring 175–372 cm in total length (TL) were sampled. Most sharks were likely immature females ranging between 200 and 260 cm TL, with few individuals < 200 cm TL being caught. This contrasts greatly with the tiger shark size-distribution previously reported for coastal waters off the Brazilian mainland, where most individuals measured < 200 cm TL. Also, the movements of 8 individuals measuring 202–310 cm TL were assessed with satellite transmitters for a combined total of 757 days (mean = 94.6 days∙shark^-1^; SD = 65.6). These sharks exhibited a considerable variability in their horizontal movements, with three sharks showing a mostly resident behavior around FEN during the extent of the respective tracks, two sharks traveling west to the South American continent, and two sharks moving mostly along the middle of the oceanic basin, one of which ending up in the northern hemisphere. Moreover, one shark traveled east to the African continent, where it was eventually caught by fishers from Ivory Coast in less than 474 days at liberty. The present results suggest that young tiger sharks measuring < 200 cm TL make little use of insular oceanic habitats from the western South Atlantic Ocean, which agrees with a previously-hypothesized ontogenetic habitat shift from coastal to oceanic habitats experienced by juveniles of this species in this region. In addition, this study adds evidence that tiger sharks are able to connect marine trophic webs from the neritic provinces of the eastern and western margins of the Atlantic Ocean across the equatorial basin and that they may experience mortality induced by remote fisheries. All this information is extremely relevant for understanding the energetic balance of marine ecosystems as much as the exposure of this species to fishing pressure in this yet poorly-known region.

## Introduction

Large-bodied shark species are often regarded as important high-level predators that regulate food webs in some aquatic ecosystems by exerting top-down effects upon the distribution, abundance and behavior of their prey, thus contributing to shape the structure of many communities [1–4]. Also, several of these species are known to perform large-scale movements across the range of their distribution [5–6], frequently synchronizing such movements with predictable food pulses associated with the life cycle of their prey [7, 8]. As a result, energy-flow between disparate, remote areas may be partially enabled due to the ecological traits of these species. Despite the potential significance of sharks in connecting and balancing marine ecosystems, alarming declines of several shark populations resulting from overfishing and habitat degradation have been reported worldwide [9–10]. Since sharks generally have *K*-selected life-history strategies characterized by slow growth, late maturity and low fecundity [11], they are typically unable to effectively replenish their populations when facing substantial anthropogenic pressure. Increasing our knowledge about the spatial ecology of large-bodied sharks and their interactions with diverse habitats and fisheries is thus necessary for understanding the trophic mechanisms that underlie several aspects of marine ecosystem dynamics and for guiding informed management policies [12].

The tiger shark, *Galeocerdo cuvier*, is the largest extant carcharhinid, growing up to 5.5 m in length [13]. It is circumglobal at tropical and warm temperate latitudes and occurs in both coastal and oceanic habitats [14–15]. Early-juvenile tiger sharks are commonly found on the shelves of continental landmasses in the western Atlantic Ocean [16–17] but they seem to undergo an ontogenetic habitat shift into oceanic waters as they grow larger [18]. It is thus possible that there is some level of size segregation in tiger shark populations across the extent of their range. Recent satellite telemetry data depicted long-distance movements, in the order of thousands of km, made by this species across the pelagic realm [5–6, 19, 20], but earlier tag-recapture data had pointed out that tiger sharks were able to perform large-scale displacements, including across ocean basins [21]. As with several other elasmobranchs [22–23], temperature-driven, seasonal latitudinal migrations are reportedly present in this poikilothermic species because tiger sharks tend to extend their distribution to higher latitudes during the warm season and return to lower latitudes during the cold season [6, 24]. Horizontal movements elicited by drivers other than temperature remain to be generally elusive as there seems to be a considerable intraspecific variability in tiger shark behavior [24–25], but predictive migrations to particular areas have been previously associated with reproductive triggers [26–27] and foraging opportunities [8–28]. Since tiger sharks are known to be generalist predators and to consume a wide diversity of *taxa* [29–30], they might benefit from using extended home ranges which include higher quantity and variety of potential prey. However, this could also imply that this species is exposed to a broader fishing pressure throughout its life span.

The tiger shark is occasionally caught as bycatch in both commercial pelagic longline fisheries [15] and coastal artisanal fisheries [31]. In recreational fisheries it is considered to be a premium species [32], but the impacts of such fisheries have been partially hindered by the establishment of non-lethal practices in game fishing tournaments [33]. Presently, the International Union for the Conservation of Nature categorizes tiger shark vulnerability to extinction as Near-Threatened (N/T) and there is evidence of significant population declines in regions where this species has been heavily harvested [32, 34]. Yet, signs of stable or even increasing abundance have been documented in areas from the Northwest Atlantic with well-regulated shark fisheries [35]. Because tiger sharks move through wide home ranges that encompass multinational, jurisdictionally-distinct waters, their effective conservation may depend on a spatially-explicit understanding of habitat use and functioning throughout the extent of their distribution [36]. Little is yet known about this species in the equatorial Atlantic Ocean, though. On that account, the present study introduces a preliminary assessment of tiger shark population structure at the insular system of Fernando de Noronha and reports large-scale movements performed by this species in this oceanic region. The hypotheses that tiger sharks using the island 1) are larger, on average, than tiger sharks using coastal waters off northeastern Brazil, and 2) move through wide distances across the equatorial pelagic environment are examined. The possible implications of tiger shark spatial distribution and behavior for species conservation are also discussed.

## Materials and methods

### Study site and sampling procedure

**Fig 1:**
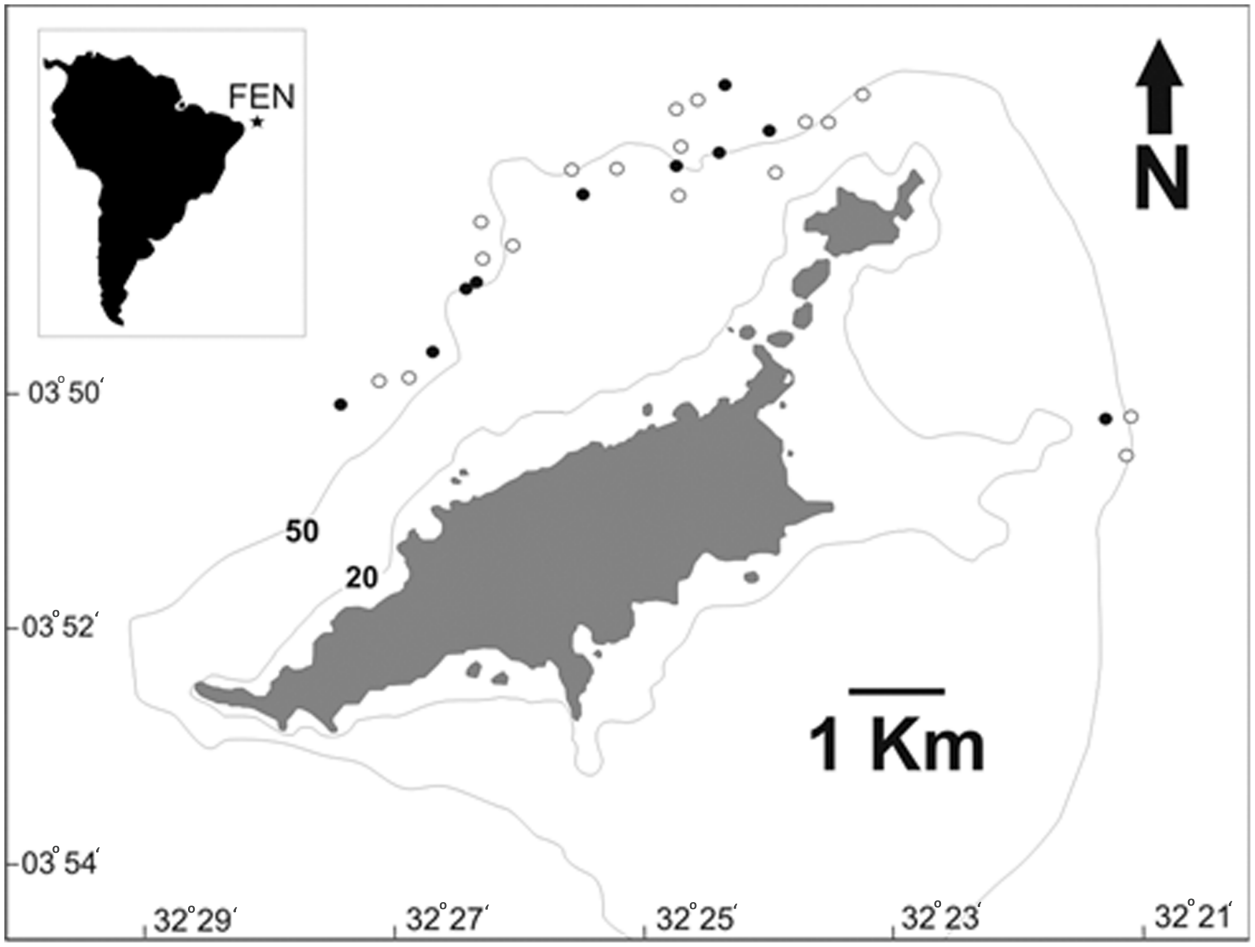
Study area. Geographical location of the Fernando de Noronha Archipelago in the western South Atlantic Ocean depicted by the star in the inset panel, and the map of the archipelago depicting the distribution of longline deployments in 2011 and 2012. Black circles correspond to sites where at least one tiger shark was captured, whereas white circles correspond to sites where no tiger shark was captured.

The Fernando de Noronha Archipelago (FEN) is an isolated group of small volcanic islands located in the western equatorial Atlantic (03°51’S; 32°25’W), 345 km off Northeast Brazil (Fig 1). This region is under the influence of the South Equatorial Current, with seawater temperature and salinity being relatively steady year-round and averaging 26°C and 36 ppt, respectively [37]. FEN is a famous no-take marine protected area in Brazil and is sought for the year-round availability of charismatic marine megafauna, most notably sea turtles, dolphins and elasmobranchs.

All sampling procedures were approved by the Committee on Ethics for the Use of Animals of the Universidade Federal Rural de Pernambuco (CEUA #23082.025519/2014) and did not involve anesthesia, euthanasia or any kind of animal sacrifice. Field work permit was issued by the Instituto Chico Mendes para a Conservação da Biodiversidade (ICMBio #43305–6).

Tiger sharks were caught with bottom longline and handline deployed off FEN between the ~30- and 65-m isobaths. The longline was used from July 2011 to August 2012 and was composed of a 275 m braided polypropylene mainline with 8 mm in diameter equipped with ten branch lines, which were attached to the mainline every 25 m with a snap. Branch lines were composed of a 5 m monofilament nylon line with 2.5 mm in diameter connected to a swivel, a wire leader 1 m in length, and an 18/0 circle hook. This gear configuration was similar to the longline gear used in a previous survey conducted off Recife, northeastern Brazil [16], thus allowing the catch composition to be compared. Longline soak time equaled ~2.5 hours and the deployment location was recorded with a handheld GPS (Garmin, USA). Bait was mostly composed of local teleost species, including *Cephalopholis fulva*, *Gymnothorax funebris* and *Caranx* spp. Posteriorly, chummed handline sets were conducted on a daily basis from dawn to dusk between 30 July and 7 August 2014. Handline gear was comprised of heavy-duty, multifilament nautical line 8 mm in diameter connected to stainless steel leaders 3.5 mm in diameter and 20/0 circle hooks, and it was used in an attempt to minimize the time spent on hook before sampling and tagging. Upon capture, the sharks were either restrained in the water alongside the boat or brought onto a hydraulic platform aboard the M/V Ocearch, emerged, eye-covered and fitted with a hose with running seawater in their mouth for ventilation. The latter procedure applied to sharks sampled in 2014 only. All sharks were sexed, measured for total length (TL) to the nearest centimeter, and tagged with a numbered, stainless steel dart tag (Floytag, USA). Some sharks were also fitted with a satellite transmitter.

Pop-up satellite archival transmitter (PSAT) tags (miniPAT; Wildlife computers, USA) were fitted to the sharks by puncturing a ~3 mm diameter hole in the proximal region of the first dorsal fin, through which a coated, stainless steel cable was inserted and crimped to the tag in such a way that the tag would be towed just posteriorly to the dorsal fin margin. PSAT-tags were programmed to archive depth, temperature and luminosity data every 3 seconds for 120 days at liberty and then to release from the sharks in order to transmit the collected data to the ARGOS satellites. Estimates of the movements performed by the sharks during the study span were based on the light-level variation throughout each day of the track, whereas the position of the shark when the tag popped-off and ascended to the sea surface was estimated with higher precision based on Doppler effect measurements conducted by the ARGOS satellites. Depth and temperature data were not addressed in this study. Additionally, fin-mounted, smart position and temperature transmitting (SPOT) tags (SPOT5; Wildlife computers, USA) were also deployed. These tags render ARGOS-based geolocation estimates each time the dorsal fin of the shark breaches out of the water and the tags successfully uplink to the ARGOS satellites, and expectedly allow tiger shark horizontal movements to be tracked more accurately. ARGOS-based geolocation estimates are classified in location classes (LC) according to their associated error, i.e. 3, 2, 1, 0, A and B, with the smallest error corresponding to LC3 estimates and the greatest error corresponding to LCB estimates [38].

### Data analysis

Tiger shark catch rate in the longline was informed using an index of catch per unit of effort (CPUE), as the number of individuals caught per 10 hooks. No attempts were made to set up a measure of abundance for tiger sharks captured with handline due to the distinct characteristics of both fishing methods deemed to be incomparable. A length-frequency distribution plot of the tiger shark catch was generated for comparisons with results reported for the Brazilian mainland [16].

Luminosity data collected by PSAT tags were used to generate daily location estimates using the manufacturer’s proprietary software (WC-GPE version 1.02.005). Raw position estimates were then filtered with an unscented Kalman filter state-space model [39] using the kftrack package in R (R Development Core Team). The more complex ukfsst package [40], which provides a similar state-space model that also incorporates a sea surface temperature (SST) feature in order to adjust the estimated daily locations in accordance with satellite-measured SST data obtained from the NOAA/ESRL database (ftp://ftp.cdc.noaa.gov), was attempted but it generally yielded unlikely geolocation estimates compared to kftrack models. The number of parameters to be estimated by the kftrack model and their respective initial values were optimized by selecting the combination of parameters and corresponding initial values that rendered the lowest negative log-likelihood among all possible combinations. Then, a bathymetric correction was applied using the analyzepsat R-package [41] and data obtained from NOAA Coastwatch (http://coastwatch.pfeg.noaa.gov) to establish a most-probable track for each tagged shark. Regarding SPOT tags, ARGOS-based geolocation estimates and associated errors were readily available upon data delivery. The raw estimates were first inspected for any unreasonable position fixes that would necessarily assume implausible swimming speeds (> 10 m/s, as a conservative threshold) between consecutive locations and these were discarded. The remaining data were then run into a Bayesian correlated random walk state-space model using the bsam R-library [42]. Average displacement speeds were calculated over 12-day periods throughout the track span of each SPOT-tagged shark because the speed assessed between consecutive locations correlated negatively with the amount of time elapsed between position estimates (Pearson’s product moment correlation r = -0.174; t = -2.946, degrees of freedom = 277, *p* = 0.003), suggesting that these estimates were likely an artifact of shark surface behavior. We used a 12-d period because this has been reported to be the average time required for tiger sharks to cross a distance greater than the error of the worst ARGOS location class [6]. Also, the displacement of each SPOT-tagged shark in relation to the tagging location was plotted against time at liberty. Finally, any possible evidence that a tagged shark could have interacted with fisheries during the track span was carefully examined. On that account, the variability in several aspects of the satellite transmission process along a track was examined, more precisely the location, quality, time, rate and amount of satellite uplinks.

## Results

**Fig 2:**
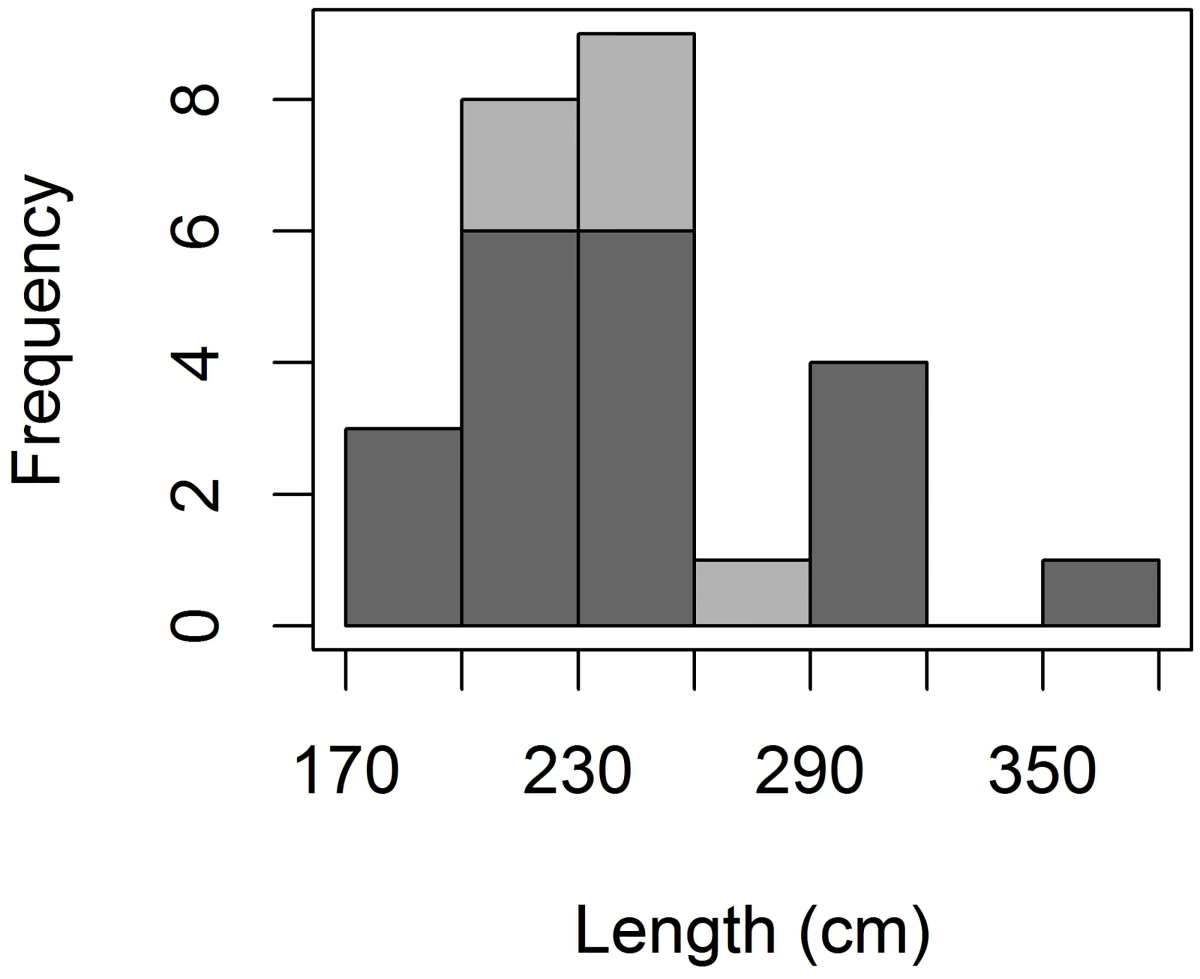
Tiger shark size composition. Total length-frequency distribution histogram of tiger sharks caught off the Fernando de Noronha Archipelago with longline and handline gear, in 30-cm size classes. The light grey and dark grey correspond to sharks caught with handline and longline gear, respectively.

A total of 29 longline sets were conducted (total effort = 290 hooks) off FEN (Fig 1), rendering the capture of 20 tiger sharks which translates into a mean CPUE of 0.69 sharks∙10 hooks^-1^ (SD = 1.14). One shark was recaptured after two days at liberty (Table 1). Tiger shark male:female ratio was 0.05:1, with only one male shark being captured. Tiger shark length ranged from 175 to 372 cm TL (mean = 244; SD = 49.8 cm TL) (Table 1). Only one female shark was considered to be adult following a size at first maturation of 315–320 cm TL [43]. Additional sampling effort conducted with handline during a nine-day period off the northern side of FEN rendered the capture of 6 juvenile tiger sharks, whose male:female ratio was 2:1 and average length was 240 cm TL (SD = 29.7) (Table 1). The length-frequency distribution histogram depicted a clear mode in shark length at the 200–230 and 230–260 cm TL size-classes, with sharks smaller than 200 cm TL being little represented in the catch composition (Fig 2). This trend was consistent regardless of whether handline data were considered or not. The overall sex ratio was 0.24 males per female.

**Table 1:** Tiger shark tag deployments.

Shark	TL	Sex	Gear	Date	Tags	Track span	Location estimates
1	310	F	Longline	02/Aug/11	C		
2	212	F	Longline	02/Aug/11	C		
3	255	F	Longline	02/Aug/11	C		
4	186	F	Longline	04/Aug/11	C		
5	260	F	Longline	04/Aug/11	C		
6	297	F	Longline	04/Aug/11	C		
7	225	F	Longline	04/Aug/11	C		
8^a^	310	F	Longline	04/Aug/11			
9	203	F	Longline	06/Aug/11	C		
10	206	F	Longline	06/Aug/11	C		
11	224	F	Longline	06/Aug/11	C		
12	199	M	Longline	06/Aug/11	C		
13	372	F	Longline	06/Aug/11	C		
14	245	F	Longline	10/Feb/12	C		
15	175	F	Longline	07/Mar/12	C		
16	212	F	Longline	01/Aug/12	C, P	125	184
17	240	F	Longline	01/Aug/12	C, P	124	193
18	238	F	Longline	02/Aug/12	C, P	10^b^	17
19	310	F	Longline	13/Aug/12	C, P	60 ^b^	40
20	260	F	Longline	13/Aug/12	C		
21	259	F	Handline	30/Jul/14	C, S	0 ^c^	0
22	251	M	Handline	31/ Jul /14	C, S	207	140
23	202	M	Handline	31/ Jul /14	C, S	123	61
24	204	M	Handline	31/ Jul /14	C, S	0 ^c^	0
25	251	M	Handline	31/ Jul /14	C, S	94	75
26	273	F	Handline	02/Aug/14	C, S	14	48

Tiger sharks captured and tagged off Fernando de Noronha, Brazil, between 2 August 2011 and 7 August 2014, with information on shark total length (TL) in centimeters, sex, the fishing gear used, date of capture, the tags deployed (i.e. conventional–C; pop-up satellite archival tag–P; and smart position and temperature transmitting tag–S), span of satellite tracks in days, and number of raw location estimates obtained.

^a^Shark #1 recaptured.

^b^Prematurely-released PSAT tags.

^c^No geolocation estimates were provided by these SPOT tags.

Four PSATs and six SPOTs were deployed in 2012 and 2014, respectively. The sex ratio of ten satellite-tagged sharks was 0.67:1 and their size averaged 244 cm TL (SD = 33.3) (Table 1). Two PSATs released from the sharks prematurely and two SPOTs failed to transmit any position fixes. For successfully-tracked sharks (*n* = 8), the collected data spanned for a combined total of 757 tracking days (PSAT = 319 days, mean = 79.8 days∙shark^-1^, SD = 55.6; SPOT = 438 days, mean = 109.5 days∙shark^-1^, SD = 79.7). PSAT tags and SPOT tags rendered 434 and 324 position estimates, respectively (Table 1).

**Fig 3:**
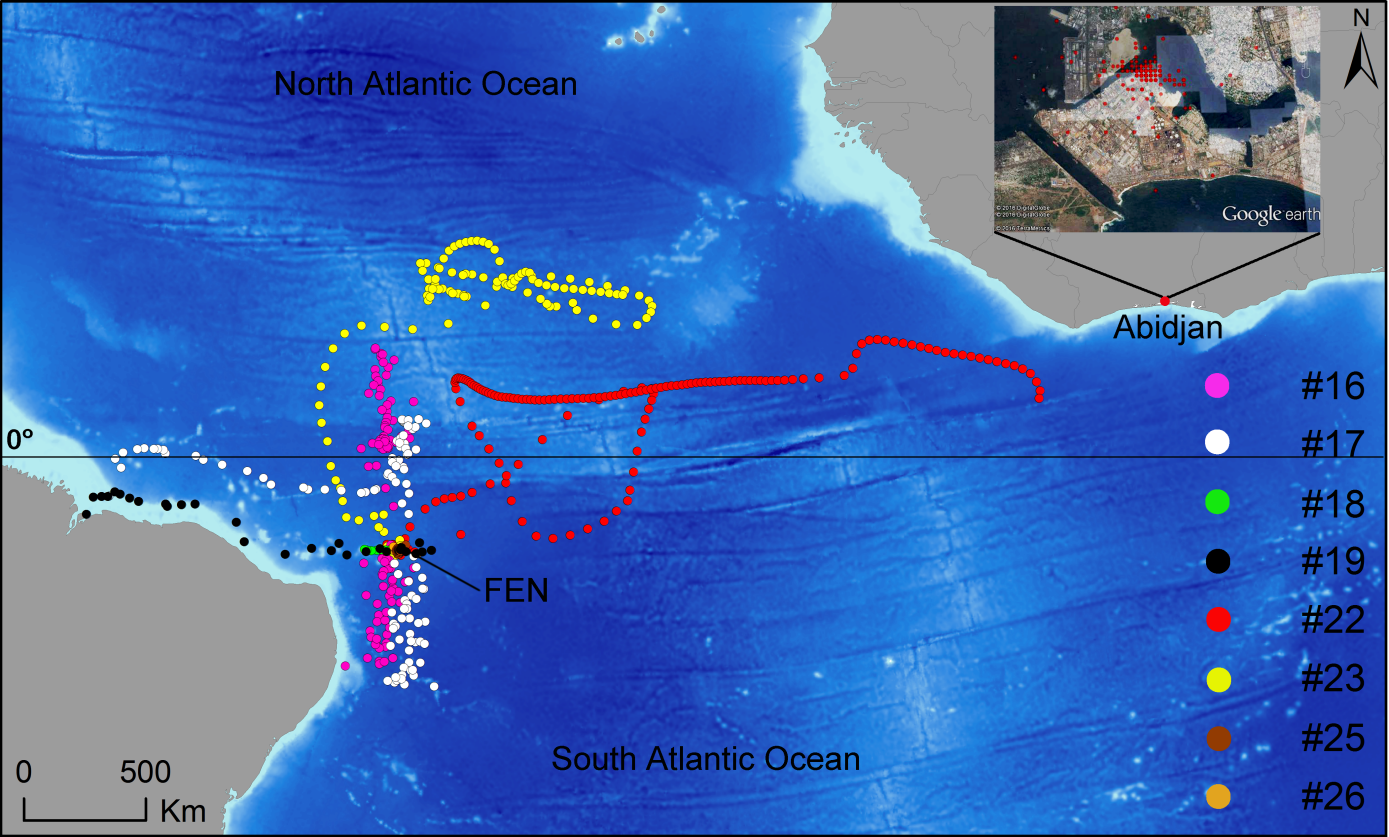
Tiger shark satellite tracks. Map depicting the most-probable horizontal movements performed by eight tiger sharks tagged with pop-up satellite archival tags (PSAT; sharks #16, #17, #18 and #19) and smart position and temperature transmitting tags (SPOT; sharks #22, #23, #25 and #26) in the equatorial Atlantic Ocean. The horizontal line represents the equator. The inset map depicts location estimates of the SPOT tag deployed in shark #22. This tag stopped providing location estimates off Liberia on 23 February 2015 but it suddenly resumed satellite transmissions on 16 November 2015, when it was located on land in Adibjan, Ivory Coast.

**Fig 4:**
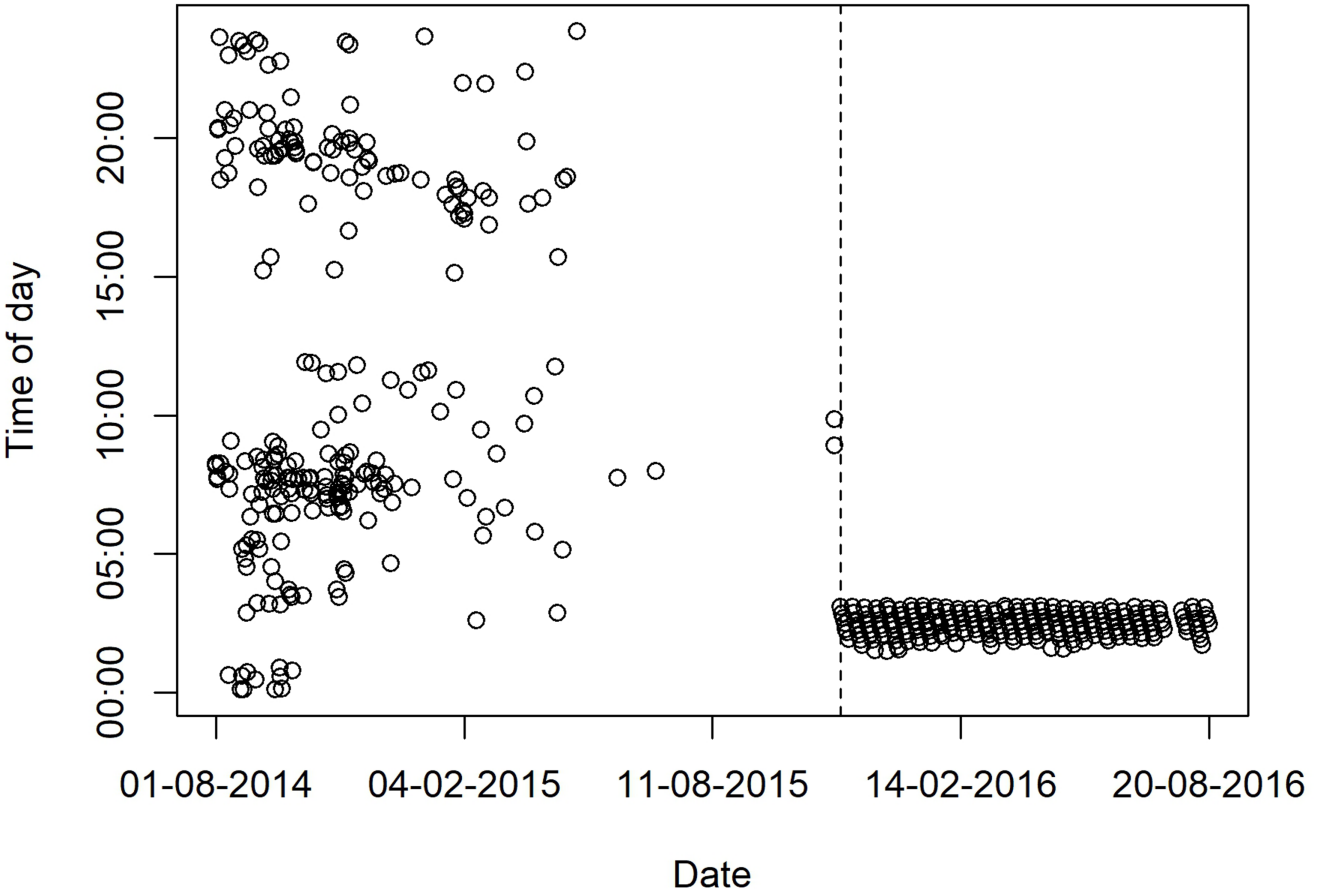
Distribution of satellite transmission time. Variation of the time at which satellite transmissions were performed along the track of tiger shark #22. The vertical dashed line depicts the first transmission from land in Abidjan, Ivory Coast, which occurred in 16 November 2015.

**Fig 5:**
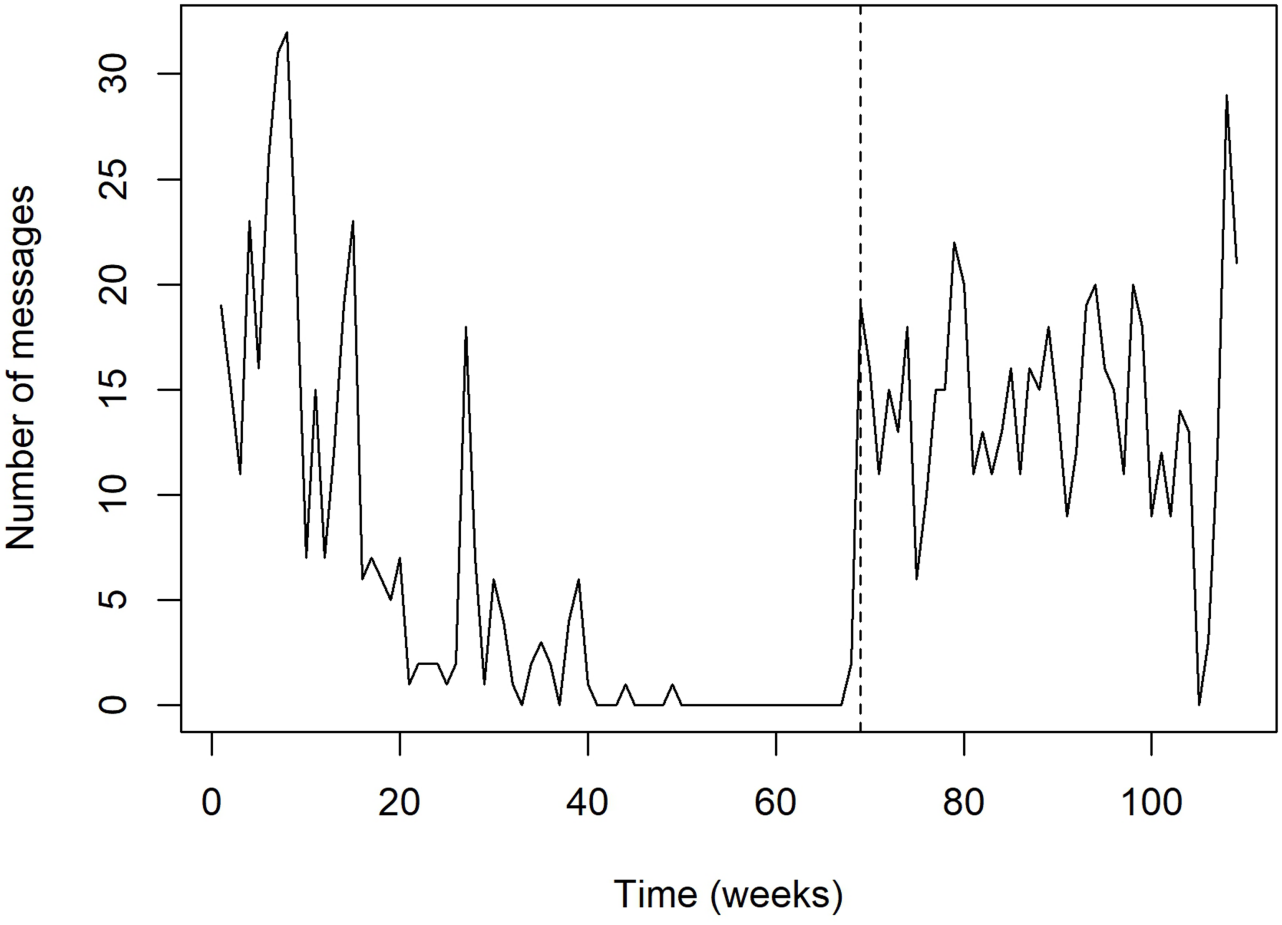
Distribution of the number of satellite transmissions. Variation of the total number of satellite-uplinked messages per week along the track of tiger shark #22. The vertical dashed line depicts the first transmission from land in Abidjan, Ivory Coast, which occurred in 16 November 2015.

The horizontal movements performed by eight satellite-tagged sharks exhibited considerable variability, with three sharks (i.e. sharks #18, #25 and #26) remaining in the vicinity of FEN for protracted periods of time and the remainder generally spending little time at liberty in this region and performing wide-ranging movements across the oceanic realm instead (Fig 3). Sharks #17 and #19 traveled west to the South American continent, shark #16 made mostly latitudinal movements to the north and south of FEN, whereas shark #23 moved promptly to the northern hemisphere along the middle of the oceanic basin soon after tagging (Figs 3 and 4). Also, shark #22 traveled east to the African continent and it was off Liberia when the tag stopped providing location estimates (Fig 3), which occurred in 207 days at liberty, even though satellite uplinks with no associated geolocation were sparsely detected for a longer period of time, until 29 June 2015 (Fig 4). However, this tag suddenly resumed rendering high-quality geolocation estimates on 16 November 2015, i.e. 474 days after the tagging date, being indisputably located on land around a Port area in the neighborhood of Port-Bouët in Abidjan, Ivory Coast (Fig 3), and kept transmitting from the same location for ~9 months. While it is unequivocal that the satellite tag was collected by humans, presumably fishers, the linearity of the most-probable track estimated by the state-space model during the wide eastward movement might render the assumption that the shark was swimming freely uncertain. However, there is evidence that the tag was not removed from the ocean before shark #22 reached the African continent. For example, a dry tag would continuously transmit messages each day from midnight until the time it reaches a maximum allowed number of messages per day, thus resulting in transmission schedules being concentrated in the first few hours of the day. Hence, the capture of a tagged shark could be revealed by a shift in transmission distribution to earlier times of the day. Such a pattern is clearly visible in the transmission schedule of tag #22, with satellite uplinks being performed around the clock until 29 June 2015 and shifting to be exclusively performed within the first ~3 hours of the day after 16 November 2015, when the tag was transmitting from land (Fig 4). The fact that two satellite uplinks were performed on 11 November 2015 around 9:00 a.m. (Fig 4) after a protracted silence might inclusively indicate that the shark was caught in this day, but this inference lacks robustness because no further evidence of the exact capture date could be found. On the other hand, and despite considerable fluctuations, the number of messages transmitted per week gradually decreased during the first eleven months at liberty, after when no transmissions occurred until the time the tag started transmitting from land, with the amount of messages exhibiting a seemingly stable trend thereafter (Fig 5). Moreover, the transmission rate of an exposed dry tag soon decreases due to the onset of a slow mode, but fast-mode transmission rates of 60 seconds indicative of tag submersion were present until November 2015. Finally, the tag rendered a much greater proportion of highest-quality (LC3) position estimates after November 2015 than during the previous period (0.175% against 0.007%, respectively). Taking all the evidence into account, it seems most likely that shark #22 was swimming freely when it moved to the eastern Atlantic Ocean and that it was caught, presumably by fishers from Ivory Coast, anytime between 29 June and 16 November 2015. The absence of satellite uplinks during this period might have resulted from a fouled conductivity sensor, with the tag resuming transmission routines after being landed and cleaned up.

**Fig 6:**
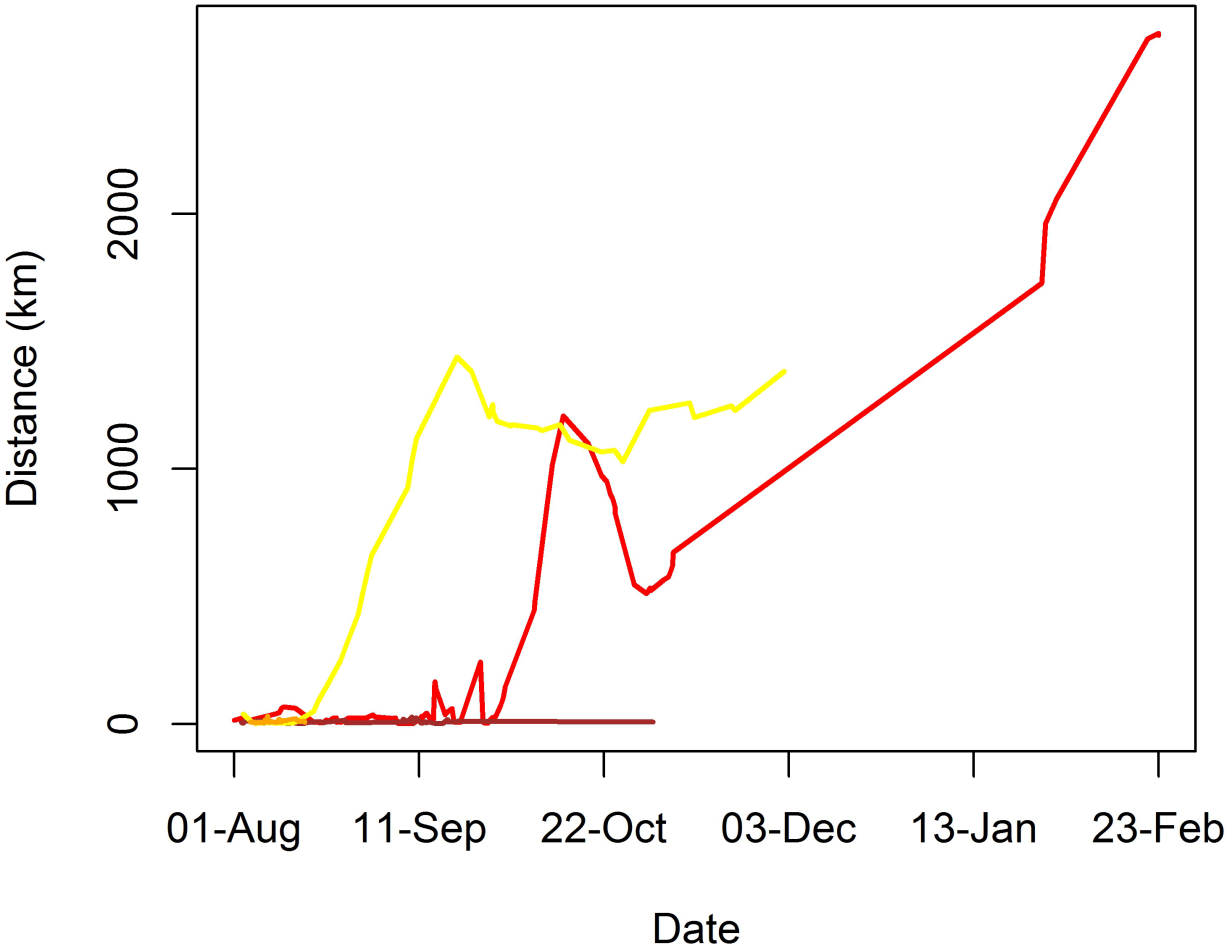
Tiger shark displacement through time. Linear distance to the tagging site of tiger sharks tagged with SPOT tags between 30 July and 2 August 2014 off the Fernando de Noronha Archipelago across the extent of their respective tracks. Red line: shark #22; yellow line: shark #23; brown line: shark #25; orange line: shark #26.

A positive relation between the distance traveled away from the tagging location and time at liberty was clearly present in two SPOT-tagged sharks (i.e. sharks #22 and #23), which performed wide-ranging movements up to nearly 3,000 km away from the tagging site (Fig 6). Conversely, two other SPOT-tagged sharks (i.e. sharks #25 and #26) showed a mostly-resident behavior during the extent of their tracks. Until the last satellite transmission while free-swimming, SPOT-tagged sharks were at large for 14–207 days (mean = 109.5; SD = 79.7) (Table 1) and moved 27–2,707 km (mean = 1,051; SD = 1,289) away from the tagging site. Overall displacement speeds over 12-d periods for SPOT-tagged sharks averaged 0.28 m∙s^-1^ (SD = 0.29; range = 0.003–1.00). As expected, the two sharks that showed higher dispersion from the tagging site (i.e. sharks #22 and #23) moved at greater average speeds (0.32 and 0.43 m∙s^-1^, respectively) than the sharks that remained close to the tagging site, whose average speeds were one order of magnitude lower (0.04–0.05 m∙s^-1^). The fact that sharks #22 and #23 moved at a similar mean speed lends further support to the hypothesis that shark #22 was swimming freely during its intercontinental movement. In regard to PSAT-tagged sharks, the distance of pop-up locations to the tagging site ranged between 146–1,296 km (mean = 705; SD = 637) and the corresponding time-at-liberty ranged between 10–125 days (mean = 79.8; SD = 55.6) (Table 1).

## Discussion

The ecological role of highly-mobile marine predators is yet to be thoroughly determined and quantified in general terms [44]. However, it is expectable that these species could play an important part in connecting geographically-distant habitats by enabling energy and nutrient flow between distinct or remote trophic webs which would otherwise be uncoupled [45–46]. Furthermore, some of these species may regulate the structure and dynamics of several marine communities across the extent of their distribution through top-down trophic effects [3–4]. In particular, apex predatory species that make use of wide home ranges encompassing disparate habitats where they prey upon a diversity of meso- to high-level consumers might be most significant to the balance of marine ecosystems [47]. The tiger shark is probably one of the best examples of these species because *i*) it is generally acknowledged to be a marine top predator, *ii*) it preys upon diverse *taxa* characteristic of disparate habitats including marine mammals, reptiles, birds, fish and cephalopods [29–30], *iii*) it performs wide-ranging, ocean-scale movements [5], and *iv*) it uses a considerable variety of marine habitats available in tropical and warm temperate latitudes, from coastal [35] to oceanic areas [15] including insular systems [26, 48], and from the sea surface down to waters > 1,000 m in depth [18, 48]. Being generalist top predators with little habitat specialization and wide home-ranges, tiger sharks could be greatly responsible for promoting the connectivity of many marine communities and for maintaining the abundance of several populations of consumers in balanced numbers. Albeit these attributes might render tiger sharks less vulnerable to human pressure compared to other elasmobranch species [49], the ecological significance of this apex predator [47] warrants the spatially-explicit understanding of tiger shark distribution and behavior to guide future conservation efforts.

Most tiger sharks sampled off FEN were likely immature females measuring 200–260 cm TL. This contrasts with the results obtained in coastal waters off the Brazilian mainland, where the bulk of the tiger shark catch corresponded to small juveniles of both sexes measuring < 200 cm TL [16]. Distinct size compositions in oceanic and coastal environments may derive from ontogenetic habitat shifts in this species during the juvenile stage. Young-of-the-year tiger sharks have been reported to occur on the continental shelves of the western Atlantic Ocean [16–17], but they seem to expand their habitat as they grow larger by moving into oceanic waters and performing increasingly deeper dives in the pelagic realm [18]. The intrinsic variability in behavioral traits throughout the life history of this species may thus explain a possible size-based segregation of tiger shark populations across large spatial scales, with larger individuals tending to explore oceanic areas and habitats which might be largely inaccessible to younger individuals purportedly attached to the neritic environment. Ontogenetic dietary shifts during the juvenile stage [29–30], together with spatial variability in prey availability, could also contribute to explain the observed differences in tiger shark size between coastal and insular habitats. Additional sampling efforts across large spatiotemporal scales are necessary to understand habitat utilization patterns of tiger sharks throughout their ontogeny. Notwithstanding, the present results suggest that juvenile tiger sharks from the western South Atlantic might disperse from their natal grounds on the continental shelf [16] into the pelagic realm, which they seem to use broadly [5, 6, 15].

According to the results herein reported, tiger sharks are able to connect the eastern and western margins of the Atlantic Ocean along the equatorial basin. Despite the low number of sharks sampled in this preliminary study precluding any quantitative assessment of transoceanic migratory behavior by this species, it has been demonstrated that tiger sharks tagged off northeastern Brazil can move across the Atlantic basin towards Africa. For the purpose of tracking animals over a wide spatial scale, the ARGOS-based geolocation process used by SPOT tags may provide highly-accurate position estimates with negligible, < 1 km error [38]. Even though the light-based geolocation method used by PSAT tags is indisputably less accurate [50], implying a significant amount of uncertainty in estimating the most probable track of PSAT-tagged sharks, the position fixes rendered by these tags after they release from the sharks is ARGOS-based as well. Therefore, PSAT tags accurately informed the location of the respective sharks when they popped-off and first up-linked to the satellites, being useful to assess the magnitude and direction of shark dispersive movements in relation to the tagging site in this study. Juvenile tiger sharks previously tracked with PSAT tags off the Brazilian mainland tended to perform dispersive movements of comparatively lower magnitude [31], which might be explained by the generally smaller size of these sharks and by their movements being largely associated with the continental landmass rather than the open ocean. In regard to the oceanic province, recent research based on onboard observer data hypothesized that equatorial latitudes could provide a connecting corridor for tiger sharks performing transoceanic migrations across the Atlantic [15], a proposition which the present results seem to corroborate. At higher latitudes, tiger shark oceanic excursions are apparently limited as they do not seem to cross the mid-Atlantic Ridge [6, 20], a trend which might derive from unfavorable temperature regimes faced by this poikilothermic species in comparatively colder offshore regions. Seawater temperature is expectedly an important factor regulating the oceanic distribution of tiger sharks since they conduct seasonal latitudinal migrations triggered by thermal cues, i.e. moving to higher latitudes in the summer and returning to lower latitudes in the winter [6, 24]. Moreover, tiger shark transoceanic movements along the equatorial Atlantic might be bidirectional rather than unidirectional (i.e. eastward from the Americas to Africa), given the complex current and counter-current system that prevails in this region [51]. In agreement, a > 300 cm TL male tiger shark which was recently tagged off Ascension Island in the eastern South Atlantic Ocean moved to Northeast Brazil within ~3 months at liberty (Ascension Island Government Conservation & Fisheries Department, pers. comm.). Further tagging efforts are necessary to effectively assess tiger shark transoceanic behavior in this region, but the results obtained so far indicate that several marine trophic webs from the neritic provinces of the American and African continents, as well as from the equatorial oceanic province, might be connected by the highly-mobile tiger shark.

Besides connecting disparate habitats from different continents, tiger sharks also seem to be affected by remote fisheries. Previous tagging data had shown that tiger sharks endure a significant fishing pressure in coastal and oceanic waters from the western South Atlantic [31]. The present study provides the first report of a tiger shark tagged off Brazil being harvested off Africa by fishers from Ivory Coast. Unfortunately, all attempts to retrieve information about the actual site of capture and the fishing gear used were unsuccessful. Yet, the collected evidence is most useful to understand the complexities surrounding the association between tiger shark spatial ecology and the exposure of this species to fishing exploitation. For example, highly-migratory pelagic sharks have been reported to use predictable hotspot habitats that overlap greatly with the distribution of oceanic longline fishing areas [52]. Ascertaining which areas and corridors are most likely to be used by vulnerable marine resources for foraging, reproducing and migrating is thus essential to implement effective fisheries management policies, particularly concerning wide-ranging species that require international conservation efforts such as the tiger shark. Recent genetic evidence suggesting that the western South Atlantic Ocean may have been a historical zoogeographic connection linking tiger shark populations from the Atlantic and Indo-Pacific via dispersal around South Africa [53] lend further importance to a possible equatorial intercontinental corridor. The extent and impacts of unreported and unregulated harvest of tiger sharks in the equatorial Atlantic Ocean are widely unknown. However, the possible significance of this region to both transoceanic and interoceanic connectivity between tiger shark populations raises concerns about the species’ exposure to fishing pressure and warrants further research on its spatial ecology at an oceanic scale.
